# Supplementary material for: Sample-specific haplotype-resolved protein isoform characterization via long-read RNA-seq-based proteogenomics
Source: bioRxiv. 2026 Mar 4:2025.11.21.689818. Preprint. [Version 2] doi: 10.1101/2025.11.21.689818 (PMC12980389; doi:10.1101/2025.11.21.689818)
Supplement: Supplement 1 [file NIHPP2025.11.21.689818v2-supplement-1.pdf]

bioRxiv preprint doi: https://doi.org/10.1101/2025.11.21.689818; this version posted March 4, 2026. The copyright holder for this preprint (which was not certified by peer review) is the author/funder, who has granted bioRxiv a license to display the preprint in perpetuity. It is made available under aCC-BY 4.0 International license.

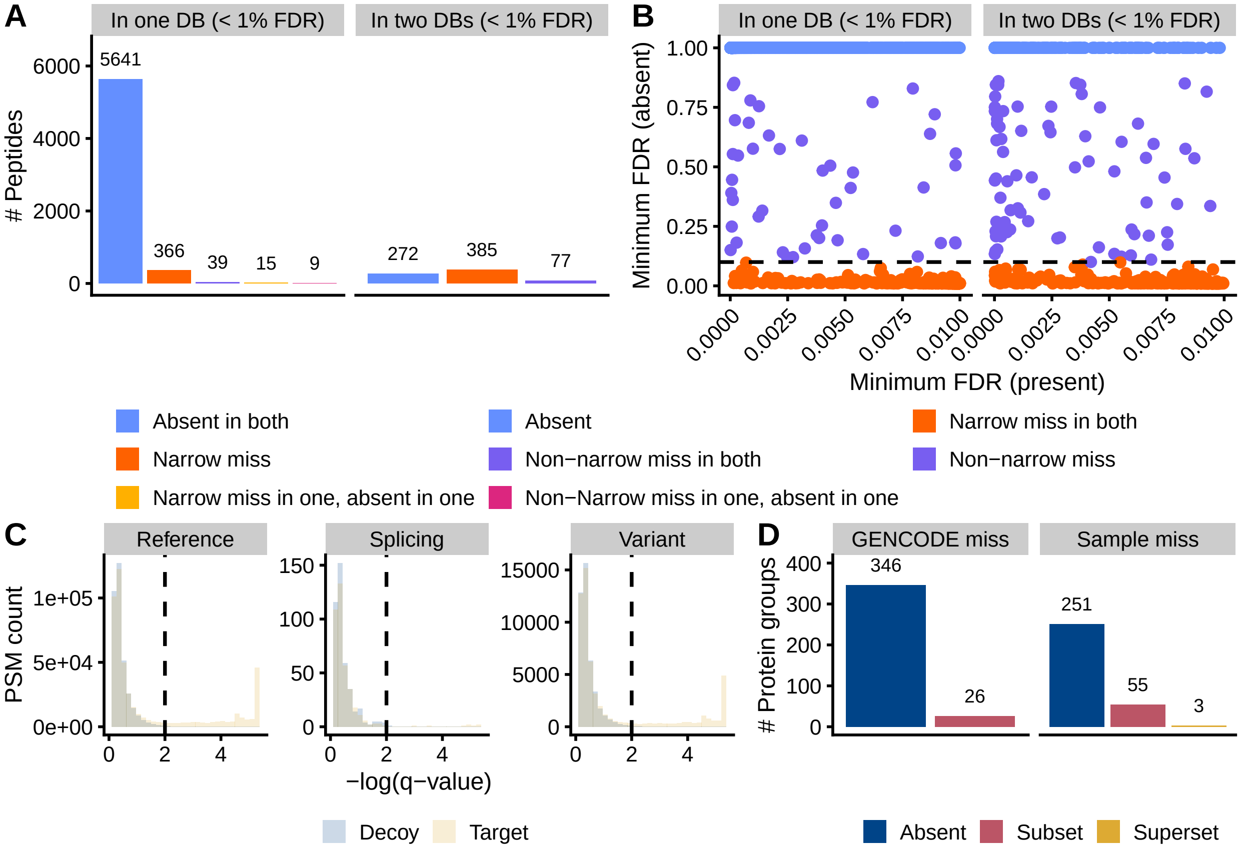

Fig. S1 Reasons for peptide differences between different databases, calibration of decoy-target FDRs in our sample-specific database, and protein group differences between our sample-specific, GENCODE-derived database and the GENCODE reference. A. Discrete classification, taking into account whether the peptide in question behaved similarly in databases in which it was not detected. **B.** Quantitative classification, using the minimum q-value for databases in which the peptide was detected (if the peptide was detected in two databases) or peptides in which the peptide was not detected (if the peptide was detected in a single database). Absent in both indicates that the peptide did not have any PSMs in the missing database(s), independent of q-value. Narrow miss in both and narrow miss indicate that the peptide had at least one PSM with a q-value $q < 0.1$ in the missing database(s). Non-narrow miss in both and non-narrow miss indicate that the peptide had at least one PSM with a q-value $q > 0.1$ in the missing database(s). Narrow-miss in one, absent in one, and non-narrow miss in one, absent in one each indicate that the peptide in question was absent in one of the missing databases and had a narrow or non-narrow miss in the other (definitions as before). **C.** Calibration of estimated q-values in the target-decoy framework on the WTC11 cell line, stratified by the source of the peptide. Only peptides mapping to a single protein isoform were considered. Peptides were assigned to the reference peptide group unless the protein isoforms they mapped to were alternatively spliced (in which case they were assigned as splicing peptides) or had at least one non-silent variant (in which case they were assigned as variant peptides). Peptides that could be counted as either variant or splicing peptides were counted as splicing peptides. The dotted line denotes $q = 0.01$. **D.** Reasons for protein group differences between the GENCODE reference proteome and the WTC11 sample-specific proteome, stratified by the database in which protein groups were missing. Absent denotes that at least one protein of the protein group was absent from all inferred protein groups for the other database. Subset indicates that the protein group in question was a subset of a protein group in the other database. Superset indicates that the protein group in question was a superset of a protein group in the other database.
